# Alterations in B and NK cells highly correlate with disease severity in children with COVID-19

**DOI:** 10.55730/1300-0144.5686

**Published:** 2023-08-10

**Authors:** Ezgi TOPYILDIZ, Neslihan EDEER KARACA, Burcu TAŞKIN, Ayşe AYGÜN, Zümrüt ŞAHBUDAK BAL, Eylem Ulaş SAZ, Nuri Zafer KURUGÖL, Necil KÜTÜKÇÜLER, Güzide AKSU

**Affiliations:** 1 Department of Pediatric Allergy and Immunology, Faculty of Medicine, Ege University, İzmir, Turkiye; 2 Department of Pediatric Rheumatology, Faculty of Medicine, Ege University, İzmir, Turkiye; 3 Department of Pediatric Infection Disease, Faculty of Medicine, Ege University, İzmir, Turkiye; 4 Department of Pediatric Emergency, Faculty of Medicine, Ege University, İzmir, Turkiye

**Keywords:** B Lymphocytes, children, COVID-19, immunology, natural killer cells, disease severity

## Abstract

**Background/aim:**

Children with coronavirus disease 2019 (COVID-19) present milder symptoms than adults and are at lower risk of hospitalization and life-threatening complications. However, the kinetics of lymphocyte subsets and serum immunoglobulins in the peripheral blood during COVID-19 infection remains unclear. In this study, it was aimed to determine the changes in hematological and immunological parameters, especially in the lymphocyte subsets, in the peripheral blood of children with different COVID-19 disease severity.

**Materials and methods:**

The study was planned as a prospective cohort and included 68 children aged 0–18 years who were admitted to Ege University Faculty of Medicine Department of Pediatrics and diagnosed with COVID-19 infection between May 2020 and December 2021. In addition to demographic characteristics, clinical findings, and severity criteria, hematological, biochemical, and immunological laboratory (T/B lymphocyte subgroups, serum immunoglobulins) results were noted and examined if there were some correlations between disease severity and the laboratory values.

**Results:**

In the study group, while 60.6% (n = 40) of the patients received treatment in the hospital, 10.6% (n = 7) needed intensive care treatment. Lymphopenia (35.3%) was more common than neutropenia (14.7%) in the COVID-19-infected children. CD19+ B cells were low in a very high percentage of patients (26.5%), and 16.2% had low levels of NK cells. Significant correlation between disease severity and CD19+lymphocytes, CD19+CD38+IgM^low^ lymphocytes, CD19+CD38+CD27^high^IgM^high^ lymphocytes, CD19+CD81+ lymphocytes (p = 0.001, p = 0.008, p = 0.014, p = 0.025, and r_s_ = 0.394, r_s_ = 0.326, r_s_ = 0.303, r_s_ = 0.280, respectively), significant inverse correlation between disease severity and absolute lymphocytes counts and CD3-CD16+CD56+ lymphocytes (p = 0.004, 0.014, and r_s_ = −0.353, r_s_ = −0.304, respectively) were observed. The percentage of hospitalized patients with low CD3 levels (15%) was significantly higher than that of the outpatients with low CD3 levels.

**Conclusion:**

As the severity of the disease increased, the CD19^+^, CD19^+^CD38^+^IgM^low^, CD19^+^CD38^+^CD27^high^IgM^high^, and CD19^+^CD81^+^ lymphocytes percentages increased, while the lymphocyte count and NK cell percentage decreased. Therefore, detecting these prognostic immunobiomarkers related to the severity of the disease may contribute considerably to management of the illness.

## 1. Introduction

The new severe acute respiratory syndrome coronavirus 2 (SARS-CoV-2) has caused a pandemic that has affected the whole world, starting in the city of Wuhan, in China’s Hubei Province, in the end of 2019 [1]. Named coronavirus disease 2019 (COVID-19) by the World Health Organization (WHO), this disease has affected more than 693 million people and caused more than 6.9 million deaths by the end of August 2023.^1^

The SARS-CoV-2 virus is highly contagious, and individuals of all ages, including children, are at risk. Although child case rates were thought to be low in the early stages of the COVID-19 pandemic [2,3], subsequent studies have shown that children and adolescents are significantly susceptible to SARS-CoV-2 infection. By the end of June 2022, of all American cases, 17.5% were in children aged 0 to 11 [4,5]. When studies with high inclusion of the pediatric age group were examined, it was seen that the most common symptoms in patients infected with SARS CoV-2 infection were fever, respiratory symptoms, and gastrointestinal symptoms [6,7]. However, children who develop a postinfectious multisystem hyperinflammatory syndrome, called multisystem inflammatory syndrome (MIS-C), have also been described [7].

It is well-known that severe illness and intensive care hospitalization rates in children are lower than in adults. In a metaanalysis by the University of Texas-San Antonio and Texas Children’s Hospital [8], which included 131 studies and 7780 children from 26 countries, the need for observation or treatment in the intensive care unit was low (3.3%), and an underlying medical problem was reported in 655 individuals. Among these patients, the majority (65%) had a suppressed immune system or a history of respiratory or heart disease [9].

There are studies showing an apparent decrease in peripheral lymphocytes in COVID-19 patients. In addition, only a few adult studies have noted changes in lymphocyte subgroups. Therefore, a detailed examination of cellular and humoral immunity during COVID-19 infection and determination of underlying disease in pediatric age group studies are still needed [10–13].

In this study, COVID-19 patients were evaluated in regard to the clinical and immunological findings and the effects of immunological changes on the prognosis of COVID-19 were determined. In addition, it was aimed to clarify the clinical significance of hematological, biochemical, and immunological changes that may help us in the diagnosis, treatment, and follow-up strategies of pediatric COVID-19 patients.

## 2. Materials and methods

### 2.1. Study design and population

The study was planned as a prospective cohort study and included 68 children aged 0–18 years who were diagnosed with COVID-19 infection at Ege University Faculty of Medicine, Department of Pediatrics, between May 2020 and December 2021. These patients were positive for COVID-19 polymerase chain reaction or SARS-CoV-2 IgM antibodies.

First, informed consent was obtained from all of the families who accepted and were informed about the study. Detailed medical histories of the patients, including their complaints, presence of concomitant disease, and whether there were any other infected members in the family, were noted. The physician responsible for them did physical examinations, and vital signs were recorded. Information on the treatments received by the patients and the complications developed during the follow-up were also recorded. The severity of COVID-19 was defined as asymptomatic, mild, moderate, severe, or critical according to the following criteria based on clinical features, laboratory tests, and chest radiographic imaging [14] and the number of patients under this classification were indicated next to them:

- Asymptomatic infection (n: 3): without any clinical symptoms or signs, normal chest imaging results, even though the 2019-novel coronavirus (nCoV) nucleic acid test result is positive.
- Mild (n=49): symptoms of acute upper respiratory tract infection, including fever, fatigue, myalgia, cough, sore throat, runny nose, and sneezing. Physical examination shows congestion of the pharynx and no auscultatory abnormalities. Some patients may have no fever or have only digestive symptoms, such as nausea, vomiting, abdominal pain, and diarrhea.
- Moderate (n=7): pneumonia, frequent fever, and cough (primarily dry cough, followed by productive cough); some may have wheezing but no obvious hypoxemia such as shortness of breath. Some patients may have no clinical signs and symptoms, but chest computed tomography shows subclinical lung lesions.
- Severe (n=1): early respiratory symptoms, such as fever and cough, may be accompanied by gastrointestinal symptoms, such as diarrhea. The disease usually progresses within 1 week, and dyspnea occurs with central cyanosis. Oxygen saturation is <92% with other hypoxia manifestations.
- Critical (n=8): children can quickly progress to acute respiratory distress syndrome or respiratory failure and may also have shock, encephalopathy, myocardial injury or heart failure, coagulation dysfunction, and acute kidney injury. Organ dysfunction can be life-threatening. Patients who need intensive care unit admittance are also included in this category.

The study was approved by the local ethics committee (Ethics no.: 20-5T/33).

### 2.2. Laboratory evaluation

The B and T lymphocyte subgroups were studied by flow cytometry at the Ege University Faculty of Medicine Pediatric Immunology Laboratory. All of the blood samples were taken randomly, via peripheral veins, at admission and only one time into ethylenediaminetetraacetic acid (EDTA) tubes.

All flow cytometric analyses were performed using Facs Canto II (Becton, Dickinson, and Company, BD Biosciences, Franklin Lakes, NJ, USA). The data in the text were given according to standardized publishing rules. A total of 10 μL of each monoclonal antibody (BD Biosciences) was added to 100 μL of prepared whole blood in the test tubes. After vortexing, the tubes were incubated for 20 min at room temperature in darkness. A total of 2 mL of lysing solution (BD FACS; BD Biosciences) was added, and the mixture was vortexed and incubated for 10 min at room temperature in darkness. The mixture was centrifuged for 5 min and the supernatant was aspirated. Then, 2 mL of BD cell wash was added to resuspend the pellet and it was centrifuged for 5 min and the supernatant was aspirated again. Next, 500 μL of staining buffer was added and then the cells were analyzed.

The monoclonal antibody reagents (mouse antihuman; Becton-Dickinson, Temse, Belgium), labeled FITC, PE, APC, and PerCP, used for staining were as follows: CD45/CD3/CD19/CD4/CD8/CD16-56 and CD3/HLA-DR for the lymphocyte panel; CD3+isotype control, CD3+TCR AB+TCR GD, and CD31+ (CD3/CD4/CD45RA/CD45RO) for the T cell differentiation; and CD19+isotype control, CD19+CD21+CD27+CD38+IgM+IgD, and CD19+CD81 for the B cell differentiation. These parameters are shown in Figures S1–S3. The list of the immunological parameters is given in Table 1.

Serum immunoglobulins (IgG, IgM, and IgA) were detected by nephelometry. In addition, other laboratory tests, such as hematological parameters (leukocyte, lymphocyte count, neutrophil, thrombocyte count, hemoglobulin, and hematocrit), acute phase reactants (C-reactive protein (CRP), erythrocyte sedimentation rate (ESR), procalcitonin, ferritin, D-dimer, and fibrinogen), biochemical findings, and imaging tests, were also collected from the existing patient files.

### 2.3. Statistical analyses

Data were evaluated using IBM SPSS Statistics for Windows 25.0 (IBM Corp., Armonk, NY, USA) by analyzing the descriptive statistics (mean, standard deviation, minimum, maximum, median) and comparing the data of the inpatient-outpatient groups using the chi squared test. Correlation between disease severity and laboratory variables was evaluated using Spearman rank-order correlations analysis. p < 0.05 was considered statistically significant.

## 3. Results

A total of 68 COVID-19 patients (n = 32 females, 47.1% and n = 36 males, 52.9%) with a median age of 102 (min–max: 1–215) months were included in the study. All of the demographic and clinical characteristics are summarized in Table 2. Considering the presence of chronic disease in the study group, 30.9% (n = 21) had a history of chronic disease, and 8.8% (n = 6) had a history of primary immunodeficiency. Most of the patients (n = 40, 60.6%) received treatment at the hospital. About 10.6% (n = 7) needed intensive care treatment. The number of patients according to the severity of the disease is given in Table 2, in detail. The percentage who had COVID-19 together with their family members was 79% (n = 54). Complications in the study group observed during follow-up were as follows: MIS-C (n = 6, 8.8%), hepatitis (n = 3, 4.4%), tracheostomy (n = 1, 1.5%), and death (n = 1, 1.5%).

The hematological parameters, acute phase reactants, biochemical findings, serum IgG, IgA, and IgM levels, and T-B lymphocyte subgroups (Table 3) of all patients were expressed as minimum, maximum, and median values. When all of the patients were evaluated, all of the median values seemed to be at almost normal ranges, except the CRP, ESR, ferritin, and D-dimer values (Table 3). Then, each patient was compared with the normal age-related levels [15,16] and it was attempted to determine low levels that predisposed them to viral infections. These findings are listed in Table 4.

Lymphopenia (35.3%) was more common than neutropenia (14.7%) in the COVID-19-infected children. IgG and IgM levels were lower than the normal age-related values in 18 of 68 patients (26.5%). CD3+ T cell lymphopenia and CD3+CD4+ T helper, and CD3+CD8+ T cytotoxic cell lymphopenia were not common, and all of them were observed in less than 15% of patients. On the other hand, similar to the serum IgG and IgM levels, CD19+ B cells were low in most of the patients (26.5%). NK cells are well-known for their antiviral activity, and 16.2% of patients had low levels. The percentages of T cell subsets, such as CD8+TCR αβ, CD8+TCR γδ, CD4+CD45+RA+ naive T cells, and CD4+CD45+RO+ memory T cells, did not show a significant decrease in the study group. CD31+CD45+RA+ recent thymic emigrant (RTE) cell evaluation may provide rapid clinical information, especially about T cell immune function [17]. The RTE cell percentage was low in 67.2% of patients compared to the reference values in a healthy pediatric population [16].

Spearman rank-order correlations were run to examine the relationships disease severity and the laboratory data (Table 5). Regarding the relationship between disease severity and the hematological parameters, there was no significant correlation between disease severity and the leukocyte, neutrophil, or thrombocyte counts. However, there were significant and negative correlations between disease severity and the lymphocyte counts, and hemoglobin and hematocrit levels (r_s_ = −0.353, r_s_ = −0.380, r_s_ = −0.388, and p = 0.004, p = 0.002, p = 0.001, respectively).

When the relationship between disease severity and acute phase reactants was analyzed, no significant correlation between the ESR, procalcitonin, and ferritin levels was recorded, but the CRP, D-dimer, and fibrinogen levels were significant biomarkers in predicting disease severity, because of a positive significant correlation (r_s_ = 0.621, r_s_ = 0.395, r_s_ = 0.459, and p < 0.001, p < 0.012, p < 0.007, respectively).

The median IgG, IgM, and IgA levels were 918, 96, and 86.5 mg/dL, respectively. There was no significant correlation between disease severity and the immunoglobulin values.

Considering the relationship between disease severity and other laboratory findings, a significant correlation between disease severity and percentages of the CD19^+^ lymphocytes, CD19^+^CD38^+^IgM^low^ lymphocytes, CD19^+^CD38^+^CD27^high^IgM^high^ lymphocytes, and CD19^+^CD81^+^ lymphocytes (r_s_ = 0.394, r_s_ = 0.326, r_s_ = 0.303, r_s_ = 0.280, and p = 0.001, p = 0.008, p = 0.014, p = 0.025, respectively) and significant inverse correlation between disease severity and the lymphocyte count and NK cell percentage (CD3^−^CD16^+^CD56^+^ lymphocytes) (r_s_ = −0.285 r_s_ = −0.304, and p = 0.022 p = 0.014, respectively) was found. However, there was no significant correlation between disease severity and the other parameters.

In addition, the percentage of hospitalized patients with low CD3 levels (15%) was significantly higher than in the outpatient group (0.0%) (p = 0.007) (Table 6).

## 4. Discussion

Due to the differences between pediatric patients and adults in the clinical and laboratory manifestations of COVID-19, clarification of the disease manifestations and course among children is essential. In addition, there is limited knowledge regarding the immune responses and their correlations with disease severity in children. In this study, CRP, D-dimer, and fibrinogen levels were found to be significant biomarkers in predicting disease severity. An increase in CD19^+^, CD19^+^CD38^+^IgM^low^, CD19^+^CD38^+^CD27^high^IgM^high^, and CD19^+^CD81^+^ lymphocytes and decrease in the lymphocyte count and NK cell percentage were correlated with severity in COVID-19-infected children.

After COVID-19 infection, most patients develop detectable serum antibodies against the viral S protein. These responses consist of initial IgM production followed by IgA and IgG increase in the circulation [18]. Similarly, in a study conducted with 35 asymptomatic children whose parents were infected with COVID-19 in Türkiye, IgG-spike values were found to be statistically higher in the group of those who were in contact with 2 or more people compared to the group of those who were in contact with a single person [19]. In the case of congenital immunodeficiency, adequate production of antibodies in the serum cannot be achieved. In this regard, patients with chronic diseases are expected to have more severe disease, mostly requiring hospitalization. In the current study group, 30.9% had a history of chronic disease, and 8.8% had a history of immunodeficiency. IgG and IgM levels were lower than the normal age-related values in 18 of 68 patients (26.5%). Similar to the findings herein, Meyts et al. [20] gathered information on 94 patients with inborn errors of immunity and SARS-CoV-2 infection, most (56%) of whom suffered from primary antibody deficiencies. In their study, about 30% of patients were treated as outpatients, 13.8% required noninvasive ventilation or oxygen administration, and 19.1% were admitted to intensive care units [20].

In a metaanalysis, Xuefeng et al. [21] examined the clinical and laboratory characteristics of pediatric patients with SARS-CoV-2 infection. Although they reported rates of 42% asymptomatic and 3% severe COVID-19, the current study revealed different results, such as 13.3% severe-critical, 82.4% mild-moderate, and 4.4% asymptomatic. In addition, 10.6% of the patients needed intensive care treatment.

In a study by Hoang et al. [9] with 7780 COVID-19 pediatric patients, 7 deaths were reported (0.09%), and 11 children (0.14%) met the inclusion criteria for MIS-C. Complications in the current patient group observed during follow-up were as follows: MIS-C (8.8%), hepatitis (4.4%), tracheostomy (1.5%), and death (1.5%).

COVID-19 causes lymphopenia by directly inducing apoptosis of lymphocytes, thymus suppression, and bone marrow impairment mechanisms [18]. Kosmeri et al. [22] suggested that lymphopenia is infrequently documented in children, possibly because of the immaturity of the immune system and lack of angiotensin-converting enzyme 2 expression in children. Similarly, in a study by Argun et al. with asymptomatic and mild pediatric patients, the lymphocyte counts were shown to be similar to those in the control group [23]. In addition, the migration of dendritic cells, monocytes, and lymphocytes from peripheric tissues to the respiratory system is associated with increased inflammatory markers [18]. In the present study, lymphopenia (35.3%) was more common than neutropenia (14.7%) in the COVID-19-infected children. Acute phase reactants revealing inflammation, such as CRP, ESR, ferritin, and D-dimer, were remarkably high in the study group (Table 3). CRP, D-dimer, and fibrinogen levels were found to be significant biomarkers in predicting disease severity (p = 0.001, p = 0.012, and p = 0.007, respectively). In a study by Xuefeng et al. [21], high CRP and D-dimer levels, and leukocytosis were the main indicators for pediatric inpatients. In another study by Hoang et al. [9], abnormal laboratory markers included serum D-dimer, procalcitonin, creatine kinase, and interleukin-6. Several studies have indicated that the absolute numbers of B cells were within the normal range in most patients during the course of COVID-19 [24]. However, other reports have suggested a decrease in B cells, especially diminished levels in severe cases [25]. Similar to the serum IgG and IgM levels in the current study, the CD19+ B cell count was low in a very high percentage of patients (26.5%) (Table 4).

Both lymphopenia and a reduced number/activity of NK cells have been correlated with a more severe COVID-19 progression. Hoang et al. reported a remarkably lower number of CD16+56+ natural killer cells (NK) in patients with MIS-C [11]. In a study by Ozkurekci et al. from Türkiye, although the difference was not significant between the groups (p = 0.09), the absolute lymphocyte count was slightly lower in the MIS-C patients as compared to the severe COVID-19 patients [26]. NK cells are well-known for their antiviral activity, and 16.2% of patients had low levels of NK cells in the present study. A significant inverse correlation between disease severity and the percentage of CD3^−^CD16^+^CD56^+^ lymphocytes (p = 0.014) was also found in a study by Wang et al. [12].

In a study by Kratzer B et al. [27], moderately lower numbers of CD3+ CD4+ CD45RA+ CD62L+ CD31+ RTE cells were observed. Similarly, the relative and absolute amounts of CD3+ CD8+ CD45RA+ CD62L+ CD31+ RTE T cells were lower; however, neither change was statistically significant [27]. Another interesting finding in their study was that patients suffering from loss of taste/smell had significantly more CD3+ CD45RA+ CD62L+ CD31+ RTEs in their blood [27]. In the current study, the RTE cell percentage was low in 67.2% of patients compared with the reference values in a healthy pediatric population [16]. No association could be found between taste/smell loss and RTE cell numbers in the present study.

A detailed study of B cell subpopulations revealed several alterations in the distribution of B cell subsets in patients with COVID-19 [25]. CD19+CD27+CD38+ cells were often increased, representing more than 30% of B cells in some cases, especially in those that were severe [25]. Similarly, significant correlations between disease severity and the percentages of CD19^+^ lymphocytes, CD19^+^CD38^+^IgM^low^ lymphocytes, CD19^+^CD38^+^CD27^high^IgM^high^ lymphocytes, CD19^+^CD81^+^ lymphocytes (p = 0.001, p = 0.008, p = 0.014, and p = 0.025, respectively) were found.

T-cell responses are essential in eliminating viral infections by directly neutralizing infected cells or instrumenting immunological memory [18]. In the study of Chen et al., the absolute number of T cells, CD4+ cells, and CD8+ cells decreased in nearly all of the patients and were markedly lower in severe cases than in those that were moderate. In another study, peripheral lymphocyte subset alteration was found to be associated with the clinical characteristics and treatment efficacy of COVID-19 patients [12]. In the same study, a decrease in CD8+ cells tended to be an independent predictor for COVID-19 severity and treatment efficacy [12]. In the current study, the percentage of hospitalized patients with low CD3 levels (15%) was significantly higher than that of the outpatients with low CD3 levels (0.0%) (p = 0.007).

In conclusion, the percentage of patients with severe presentation and MIS-C was higher than that reported in the literature. In addition, the finding that lower NK cell and CD3 levels were correlated with more severe COVID-19 progression was similar to previous reports. Thus, a more thorough description of the immune mechanisms activated by COVID-19 infection and the detection of prognostic immuno-biomarkers related to the severity of the disease may positively affect the management of the illness and prevent severe clinical manifestations or reinfection.

## Supplementary Information

Figure S1 Representative gating for the lymphocyte subsets.

Figure S2 Representative gating for the T cell differentiation.

Figure S3 Representative gating for the B cell differentiation.

## Figures and Tables

**Table 1:** List of the immunological parameters (serum immunoglobulins and T and B lymphocytes and their subsets).

Serum IgG (mg/dL)	CD19+CD27−IgM+IgD+	CD3+HLA−DR
Serum IgM (mg/dL)	CD19+CD21^low^	CD8+TCR αβ
Serum IgA (mg/dL)	CD19+CD27+	CD8+TCR Ɣδ
CD3+	CD19+CD27+IgM−IgD−	CD4+CD45RA+
CD19+	CD19+CD27+IgM+IgD+	CD4+CD45RO+
CD3+CD4+	CD19+CD38+CD27^high^IgM^high^	CD31+CD45+RA
CD3+CD8+	CD19+CD38+CD27^high^IgM^low^	
CD3-CD16+CD56+	CD19+CD38+IgM^high^	

**Table 2:** Demographic and clinical characteristics of the study group.

n=68 (%)	

Sex (female/male)	32/36 (47.1/52.9)

Age (months) (min–max (median)/mean ± SD)	1–215 (102)/102.3 ± 72.3

Chronic disease (if any)	21 (30.9)
Primary immunodeficiency	6 (8.8)
- Di-George syndrome	1 (1.5)
- APECED syndrome	1 (1.5)
- Unclassified hypogammaglobulinemia	4 (2.9)
Secondary immunodeficiency (use of immunosuppressants)	4 (5.8)
Other (Wilson’s disease, metabolic disorders, etc.)	11 (16.0)

Presence of COVID-19 infection in the family	54 (79)

Presenting symptom	
Fever	43 (63.2)
Respiratory system symptoms	27 (39.7)
Gastrointestinal symptoms	17 (25)

Hospitalization	40 (60.6)

Follow-up in the intensive care unit	7 (10.6)

Disease severity	
Asymptomatic	3 (4.4)
Mild	49 (72.1)
Moderate	7 (10.3)
Severe	1(1.5)
Critical	8(11.8)

Complications	11 (%)
MIS-C	6 (8.8)
Hepatitis	3 (4.4)
Tracheostomy	1 (1.5)
Death	1 (1.5)

**Table 3:** Hematological, biochemical values, and immunoglobulin and T and B lymphocyte subset levels of the study group.

	Study group min–max (median) (n= 68)	Normal values for the same age group (min–max) (median)
Leukocyte count (10^3^/μL)	0.19–28.12 (6.94)	4.5–13.5 (8.2)
Lymphocyte count (10^3^/μL)	0.17–8.74 (1.79)	1.5–6.5 (2.50)
Neutrophil count (10^3^/μL)	0–21.03 (3.37)	1.8–8 (4.77)
Hemoglobulin (g/dL)	7.6–17 (12)	11.5–14.5 (12.0)
Hematocrit (%)	8.7–50 (36.2)	33–41 (36.1)
Thrombocyte count (10^3^/μL)	8–796 (250)	150–450 (273)
CRP (mg/L)	**0.3–357 (5)** *	0–5 (0.6)
ESR (mm/h)	**8–122 (34)** *	1–15 (4)
Procalcitonin (μg/L)	0.04–3.24 (0.13)	0.0–0.5 (0.5)
Ferritin (μg/L)	**19–1597 (218.5)** *	7–140 (23)
D-dimer (μg/L)	**207–4580 (938)** *	50 –550 (200)
Fibrinogen (g/dL)	99–679 (376)	175–400 (240)
IgG (mg/dL)	140–1550 (918)	646–1620 (1088)
IgM (mg/dL)	18–379 (96)	33.7–257 (104)
IgA (mg/dL)	26–372 (86.5)	54–268 (124)
CD3+ (%)	36.6–86.9 (71)	57–81 (68)
CD19+ (%)	0–59.2 (13.7)	10–27 (17)
CD3+CD4+ (%)	18.3–62.3 (41.5)	24–47 (36)
CD3+CD8+ (%)	7.7–50.6 (22.4)	17–37 (24)
CD3−CD16+CD56+ (%)	1.2–48.8 (12.3)	8–28 (14)
CD8+TCR αβ (%)	68.2–99.5 (90.6)	NA
CD8+TCR Ɣδ (%)	0.4–29.2 (8.7)	NA
CD4+CD45RA+ (%)	1.3–66.9 (29.2)	17–40 (26)
CD4+CD45RO+ (%)	1.5–45.6 (18.5)	9–23 (16)
CD31+CD45+RA (%)	4.3–65.2 (30.6)	NA
CD19+CD27−IgM+IgD+ (naive B lymphocyte)	5–97 (81)	64–84 (76)
CD19+CD21^low^ (CD21 low B lymphocyte)	1–21 (3)	2–7 (3)
CD19+CD27+ (memory B lymphocyte)	1–38 (10)	5–17 (9)
CD19+CD27+IgM−IgD− (switched memory B lymphocyte)	0–22.4 (4.3)	6–16 (9)
CD19+CD27+IgM+IgD+ (natural effector B lymphocyte)	0.5–21.3 (5.6)	4.98–27.6 (12.9)
CD19+CD38+CD27^high^IgM^high^ (plasmablast)	0–53 (10)	2–11 (6)
CD19+CD38+CD27^high^IgM^low^ (Class-switched plasmablast)	0–81 (42)	NA
CD19+CD38+IgM^high^ (transitional B lymphocyte)	0–73 (7)	8–21 (13)

* Median value is higher than the age-related normal value.

**Table 4:** Percentages of patients with low, high and normal immunological values based on normal values for age.

n= 68 (%)	Low levels	High levels	Normal levels
Leukocyte count	17 (25%)	6 (8.8%)	45 (66.2%)
Lymphocyte count	**24 (35.3%)**	0 (0%)	44 (64.7%)
Neutrophil count	10 (14.7%)	11 (16.2%)	47 (69.1%)
IgG	**18 (26.5%)**	4 (5.9%)	39 (54.7%)
IgM	**18 (26.5%)**	9 (13.2%)	35 (51.5%)
IgA	11 (16.2%)	18 (26.5%)	33 (48.5%)
CD3+ (%)	6 (8.8%)	6 (8.8%)	55 (80.9%)
CD19+ (%)	**18 (26.5%)**	2 (2.9%)	47 (69.1%)
CD3+CD4+ (%)	9 (13.2%)	9 (13.2%)	49 (72.1%)
CD3+CD8+ (%)	5 (7.4%)	3 (4.4%)	59 (86.8%)
CD3−CD16+CD56+ (%)	**11 (16.2%)**	5 (7.4%)	51 (75%)
CD8+TCR αβ (%)	2 (3%)	-	65 (97%)
CD8+TCR Ɣδ (%)	-	45 (67.2%)	22 (32.8%)
CD4+CD45+RA+ (%)	7 (10.4%)	11 (16.4%)	49 (73.1%)
CD4+CD45+RO+ (%)	9 (13.4%)	1 (1.5%)	57 (85.1%)
CD31+CD45+RA+ (%)	**41** (**67.2%)**	-	20 (32.8%)

**Table 5:** Correlation between disease severity and all laboratory data.

n= 68 (%)					
	Disease severity			Disease severity	
	r_s_	p-value		r_s_	p-value
Leukocyte count	0.083	0.507	CD3+CD4+ (%)	0.002	0.988
Lymphocyte count	**−0.353**	**0.004**	CD3+CD8+ (%)	−0.159	0.207
Neutrophil count	0.232	0.061	CD3−CD16+CD56+ (%)	**−0.304**	**0.014**
Hemoglobulin	**−0.380**	**0.002**	CD3+HLA−DR+ (%)	−0.132	0.298
Hematocrit	**−0.388**	**0.001**	CD8+TCR αβ (%)	0.182	0.148
Thrombocyte count	−0.199	0.109	CD8+TCR Ɣδ (%)	−0.163	0.194
CRP	**0.621**	**0.000**	CD4+CDRA+ (%)	0.053	0.674
ESR	0.429	0.164	CD4+CDRO+ (%)	0.013	0.919
Procalcitonin	0.292	0.148	CD31+CD45+RA (%)	0.106	0.424
Ferritin	0.431	0.051	CD19+CD27+IgM^high^ (%)	0.030	0.810
D-dimer	**0.395**	**0.012**	CD19+CD27+IgM^low^CD20+ (%)	0.059	0.639
Fibrinogen	**0.459**	**0.007**	CD19+CD21^low^ (%)	−0.175	0.163
IgG	−0.007	0.959	CD19+CD38+ (%)	−0.085	0.502
IgM	0.008	0.950	CD19+CD38+CD27^high^ (%)	−0.126	0.316
IgA	0.023	0.866	CD19+CD38+CD27^high^IgM^high^ (%)	**0.326**	**0.008**
Lymphocyte (%)	**−0.285**	**0.022**	CD19+CD38+CD27^high^IgM^low^ (%)	0.132	0.294
CD3+ (%)	−0.190	0.130	CD38+IgM^high^ (%)	0.088	0.485
CD19+ (%)	**0.394**	**0.001**	CD38+IgM^low^ (%)	**0.303**	**0.014**
			CD19+CD81+ (%)	**0.280**	**0.025**

**Table 6:** Lymphocyte subsets and serum immunoglobulins in the outpatients and inpatients.

	Inpatient n= 40 (%)		Outpatient n= 26 (%)		p-values
	Low levels	Normal levels	Low levels	Normal levels	
Leukocyte	12 (30)	22 (55)	5 (19.2)	21 (80.8)	0.045
Lymphocyte	18 (45)	22 (55)	6 (23.1)	20 (76.9)	0.070
Neutrophil	**7 (17.5)**	22 (55)	**3 (11.5)**	23 (88.4)	**0.006**
IgG	14 (35)	19 (47.5)	3 (11.5)	19 (73)	0.063
IgM	12 (30)	20 (50)	6 (23)	13 (50)	0.840
IgA	9 (22.5)	20 (50)	2 (7.6)	11 (42.3)	0.119
CD3+ (%)	**6 (15)**	27 (67.5)	**0 (0)**	26 (100)	**0.007**
CD19+ (%)	9 (22.5)	28 (70)	8 (30)	18 (69.2)	0.427
CD3+CD4+ (%)	6 (15)	26 (65)	3 (11.5)	21 (80.7)	0.410
CD3+CD8+ (%)	**5 (12.5)**	31 (77.5)	**0 (0)**	26 (100)	**0.048**
CD3−CD16+CD56+ (%)	10 (25)	26 (65)	1 (3.8)	23 (88.4)	0.069

## Footnotes

1 World Health Organization (2023). Coronavirus disease (COVID-19) [online]. Website: https://www.who.int/publications/m/item/weekly-epidemiological-update-on-covid-19---17-august-2023 [accessed 21 08 2023].
