# Supplementary figures and images for: Alterations in B and NK cells highly correlate with disease severity in children with COVID-19
Source: Turk J Med Sci. 2023 Aug 10;53(5):1205–13. doi: 10.55730/1300-0144.5686 (PMC10763789; doi:10.55730/1300-0144.5686)

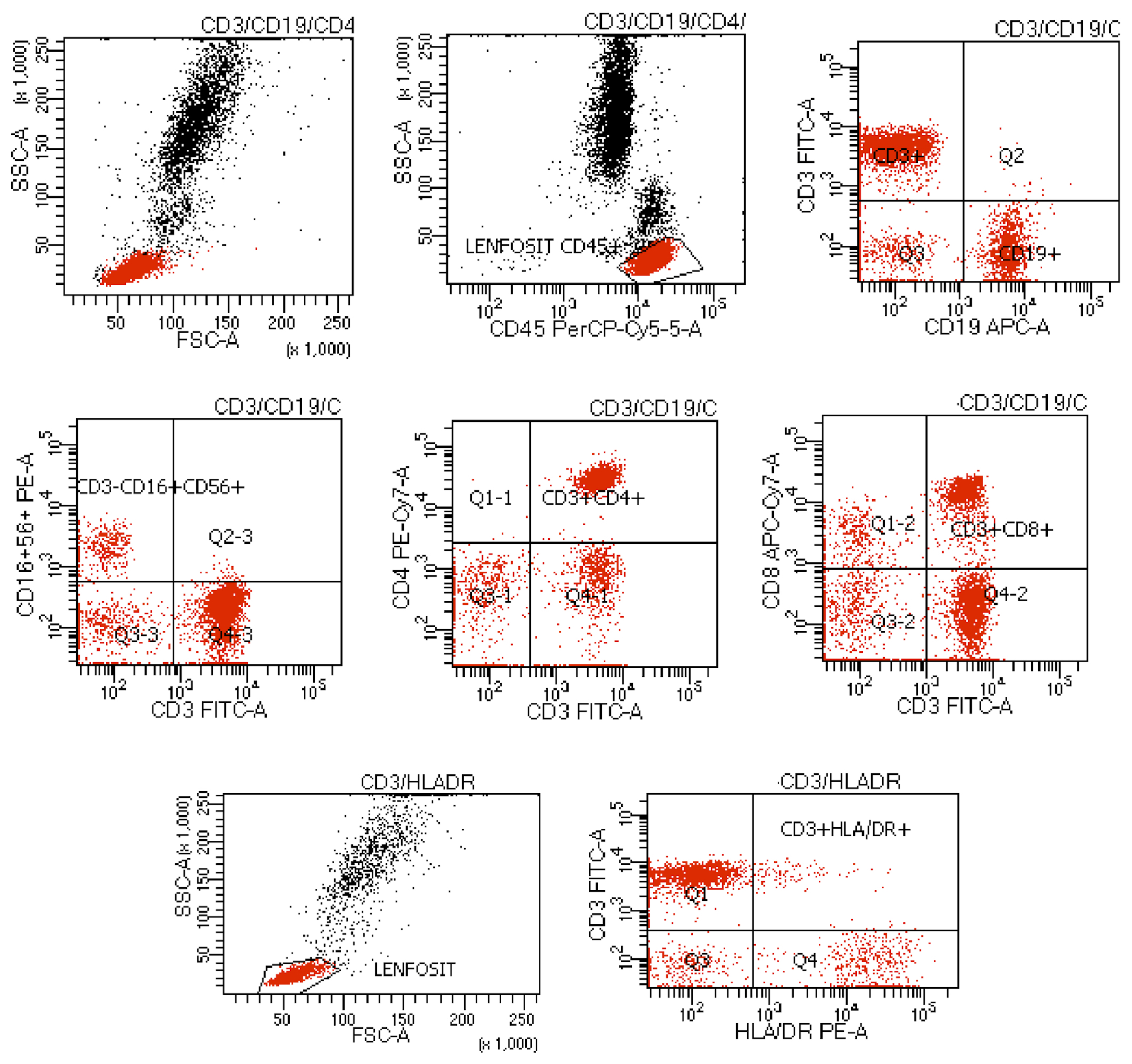

Supplement: Figure S1 — Representative gating for the lymphocyte subsets. [file turkjmedsci-53-5-1205s1.tif]

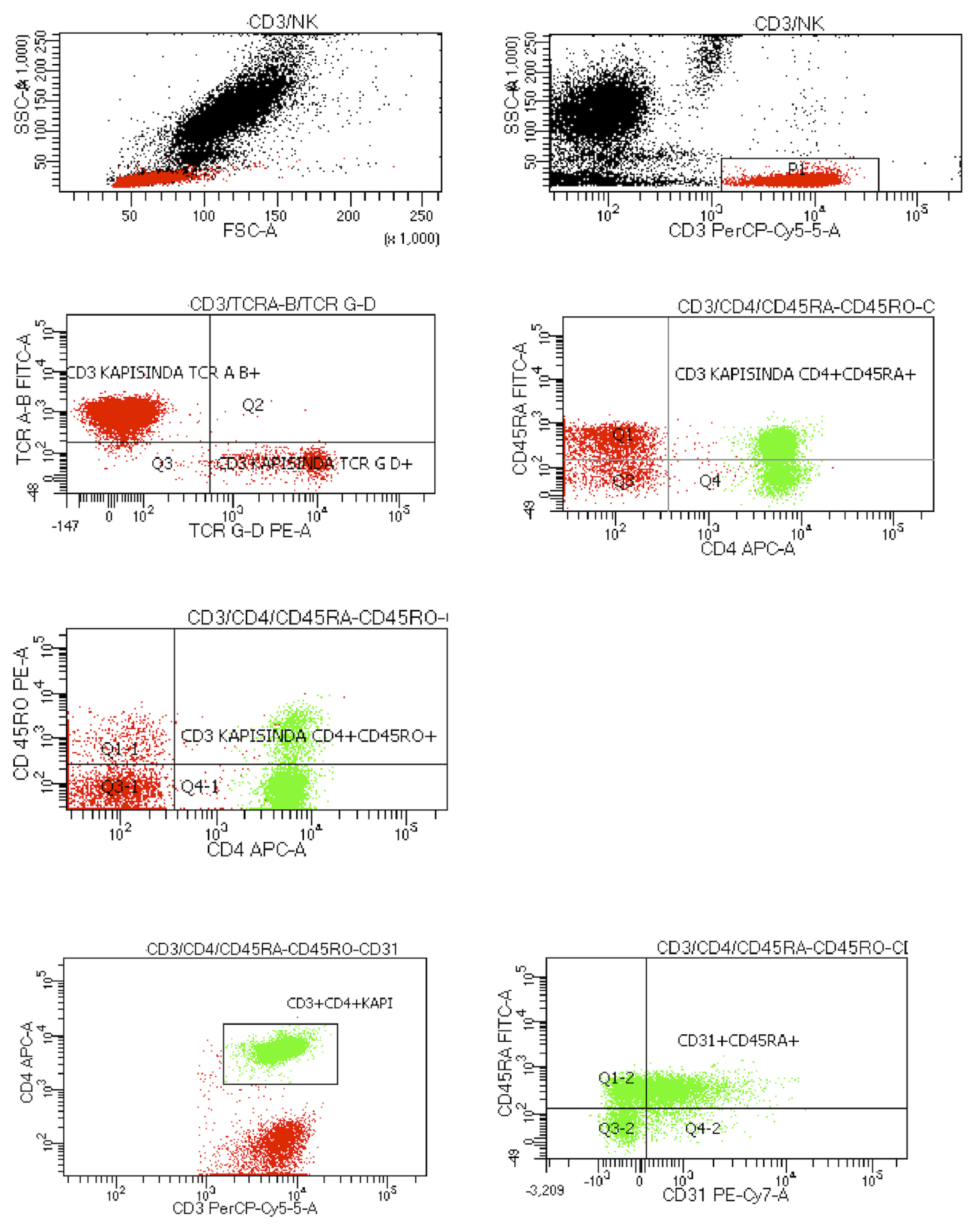

Supplement: Figure S2 — Representative gating for the T cell differentiation. [file turkjmedsci-53-5-1205s2.tif]

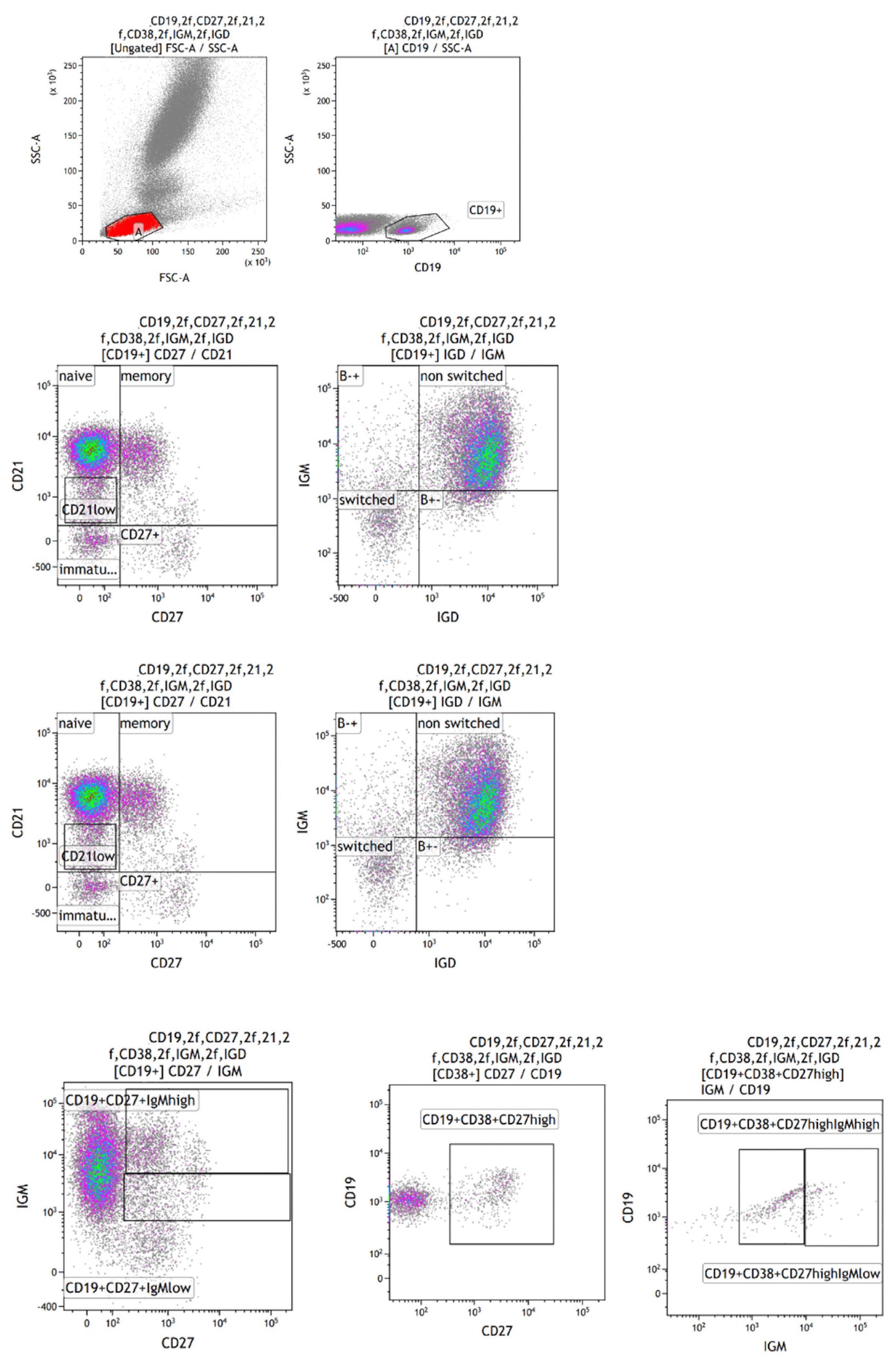

Supplement: Figure S3 — Representative gating for the B cell differentiation. [file turkjmedsci-53-5-1205s3.tif]
